# Advancing health-enhancing physical activity at workplace: Sport4Heath 2020 scientific forum

**DOI:** 10.1186/s12919-020-00196-y

**Published:** 2020-11-30

**Authors:** Nikola Todorovic, Valdemar Stajer, Bojana Harrison, Darinka Korovljev, Neboja Maksimovic, Sergej M. Ostojic

**Affiliations:** Faculty of Sport and Physical Education, University of Novi Sad, Lovcenska 16, Novi Sad, 21000 Serbia

**Keywords:** Health-enhancing physical activity, SPORT4H, Active lifestyle, Workplace

## Abstract

Physical activity at workplace can positively impact various wellbeing outcomes yet developing and implementing exercise programs that are straightforward, time-efficient and widely applicable remains a notable public health challenge. Sport4Health Network (SPORT4H) project co-funded by the European Union Erasmus+ programme unites health and sport professionals in an effort to encourage participation in physical activity among working population and reduce health risk factors for lifestyle diseases. A two-day SPORT4H scientific forum on non-traditional types of work-place exercise interventions was organized from 14th to 15th September 2020, to critically evaluate evidence on stretching and resistance exercise programs targeted to working population in aim to identify knowledge gaps and future areas of research and application. Evidence on traditional interventions (e.g., walking initiatives, active travel) appears more robust while only few studies evaluated the applicability of non-traditional PA programs in working population. However, we identified a moderate-to-strong link between non-traditional PA programs at the workplace and several health-related physical fitness indices, with resistance exercise turned out to be superior to other exercise interventions analyzed. It appears that low-volume high-repetition resistance exercise favorably affects musculoskeletal disorders, work performance and health-related quality of life in employees who exercised at least 3 times per week for over 8 weeks. In terms of safety, screening protocols should employ health-related questionnaires, adopting a progressive training load, and prescribing training programs to individual participants’ needs. Implementing non-traditional PA programs aimed to improve health-related physical fitness and counteract sedentary behavior at workplace might be therefore of utmost importance to contribute to health promotion in this sensible population.

## Background

A growing number of people spend their time sedentary and do not comply with global recommendations on physical activity (PA) for health [1], with workforce particularly susceptible to the general lack of PA. For instance, workers spend around 70% of their working hours sitting [2], and only 13% of Europeans exercise or engage in other PAs at work, while around two-thirds spend between 2.5 and 8.5 h per day sitting [3]. Physical inactivity is associated with many adverse health consequences in employees, including the increased risk of metabolic disorders, cardiovascular disease, weight gain, and type 2 diabetes [4–7]. Involvement in exercise programs leads to better health overall, significant reductions in endocrine disease and gastrointestinal prescription drug costs [8]. According to the World Health Organization (WHO), 150 min per week of moderate PA (or 75 min/week of vigorous-intensity PA, or the equivalent of a mixture of these two activities) has been recognized as a minimum dosage of activity that needs to be archived for overall health benefits [9], a recommendation that should be applied to work population as well. This only represents a minimal requirement, with additional engagement could contribute even more to subjects’ health and well-being. Since the most adults spend around 8 h per day at workplace, this perhaps opens an window of opportunity to develop effective, safe and collective workplace exercise programs.

Offering general PA programs at the workplace appears to be an effective way to enhance the levels of activity and promote health, while tackling low back pain, various muscular disorders, social issues, poor productivity, and health outcomes in workforce [10–14]. A previous review by Abdin and co-workers [15] investigated the effectiveness of various aerobic exercises for improving well-being in working adults. The authors reported mixed evidence yet it appears that workers improved their psychological well-being by participating in any form of PA. Nathan and colleagues [16] corroborated mild-to-moderate effects for walking and low-intensity workplace activities, with a combination of these activities and educational/nutritional intervention being particularly effective. However, there is a paucity of studies examining the effectiveness of so-called non-traditional PA interventions, including resistance exercise and stretching in employees.

### Sport4HealthNetwork project

Sport4HealthNetwork (SPORT4H) is a project co-funded by the European Union Erasmus+ programme that joins together health and exercise professionals from six European countries (Serbia, Belgium, Slovenia, Bulgaria, Netherlands and Croatia). SPORT4H strives to encourage participation in sport and PA among employees all around Europe to reduce health risk factors for lifestyle diseases (see: https://sport4healthnet.eu/). The project SPORT4H aims at creating better access and more opportunities in people’s everyday lives to engage in exercise and maintain a healthy lifestyle. The overall idea evaluated in this trans-national multi-year project is that advanced practices and knowledge on non-traditional PAs in the workplace represents a quantifiable health benefit, contributing to increasing healthy lifestyle behavior in working population, resulting in mood improvement, higher productivity, decrease in absenteeism and lifestyle diseases. SPORT4H ultimately leads to more specific and effective guidelines for PA promotion that should facilitate favorable behavior modification for active healthy living in the working population, and also influences stakeholders, including particularly local authorities and employers, to improve provision for this type of activity, like facilities, space and time during working hours. In aim to critically evaluate evidence on non-traditional exercise programs targeted to working population and identify knowledge gaps and future areas of research and application, we organized a two-day scientific forum on non-traditional types of work-place exercise interventions. This scientific forum was organized from 14th to 15th September 2020 in Novi Sad, Serbia, and brought together public health scientists, exercise professionals, policy administrators, and program managers. Specifically, we overviewed here the effectiveness of exercise interventions that include resistance exercise and stretching at the workplace, and assess the feasibility and safety of these alternative exercise interventions.

### Non-traditional PA programs at workplace

It appears that the working population often suffers from poor muscular fitness and musculoskeletal pain, affecting the backbone, neck, shoulders, and hips [17, 18]. Symptoms such as low back pain have been rather prevalent due to sedentary lifestyles, and prolonged-time workers spent sitting at their offices [19]. With this in mind, exercise that improves muscular fitness and joint flexibility may positively affect work performance and decrease musculoskeletal pain in this population. For example, employees with chronic pain and disability who were subjected to upper-body resistance exercise during 10 weeks successfully managed chronic pain and disability [20]; participants were physical workers who were often exposed to forceful and repetitive job tasks. In addition, stretching exercise increased range of motion, reduced back pain, and increased work performance [21, 22]. Finally, 12-week progressive high-intensity resistance exercise significantly reduced neck and shoulder pain among industrial workers [23].

Designing effective PA programs requires rather carefull analysis of non-traditional exercise administered. For example, 12 weeks of specific resistance exercise (e.g., shoulder press, lateral dubmell press, pull downs) increased maximal muscular strength and decreased perceived fatigue for regular professional activities in welding workers [24]. This kind of intervention targeted particularly delicate muscles required for optimal work performance. On the other side, general wellness programs did not affect work performance, health-related quality of life, blood lipids, blood pressure, and absentsism [25]. However, participants reduced their body weight and were more adherent to exercise. Twelve weeks of resistance or aerobic exercise minorily affected muscular fitness in construction workers, yet an improvement in aerobic capacity has been noted [26]. A handful of studies reported assorted effects of non-traditional interventions on health-related quality of life, blood pressure, lipid profile and work performance index [25–28], with a detailed list of various resistance and stretching programs presented in Table 1.

**Table 1:** Summary of studies describing non-traditional physical activity (PA) programs at workplace

Ref.	*n*	Duration	Exercise program	Measured variables	Outcomes
[29]	204 (M + F)	16 weeks	I - Progressive resistance exercise C - Bodyweight and elastic band exercises	Musculoskeletal pain intensity Blood pressure 1-RM strength BMI	I ↓; C ↓ I ↓; C ↓ I ↑; C ↑ I ↓; C ↓
[30]	350 (M + F)	12 weeks	I - Ergonomics and neck/shoulder strengthening exercises C - Ergonomics and health promotions	Work ability index	I →; C →
[27]	8143 (M + F)	72 weeks	I - Wellness program including PA and nutrition C – No treatment	Weight loss Engagement to exercise Health-related questioners Blood pressure Blood lipids Absentsism from work Job performance	I ↓; C → I ↑; C → I →; C → I →; C → I →; C → I →; C → I →; C →
[31]	142 (M + F)	24 weeks	I - Stretching exercise EG - Ergonomic modification group IE - Combined exercise and ergonomic modification group C - No treatment	Neck pain Shoulders pain Lower back pain	I ↓; IE ↓; EG ↓; C → I ↓; IE ↓; EG ↓; C → I ↓; IE ↓; EG ↓; C →
[27]	35 (M + F)	7 weeks	I - Neck shoulders resistance exercise C - Stretching and postural exercise	Pain intensity and disability Active ROM Muscular endurance SF-38	I ↓; C ↓ I ↑; C ↑ I ↑; C → I →; C →
[22]	100 (M + F)	6 weeks	I - Pelvic control hamstring stretching E - General hamstring stretching C - No treatment	Oswestry disability index Visual analog scale Work ability index Sit and reach test Straight leg raise	I ↓; E ↓; C ↓ I ↓; E ↓; C → I ↑; E ↑; C → I ↑; E ↑; C → I ↑; E ↑; C →
[32]	200 (F)	10 weeks	I - Strength training at work C - Strength training at home	Vitality and mental health (SF-36) Psychosocial work environment Work- and leisure disability Pain Scale	I ↑; C → I →; C → I →; C → I ↓; C →
[19]	96 (M + F)	24 weeks	I - Supervised strength exercise for the back and core muscles while on duty C - No treatment	Back and core muscular endurance	I ↑; C →
[24]	14 (M)	12 weeks	I – 60 min of specific (welding) strength training C - No treatment	1RM strength Blood pressure RPE during workdays	I ↑; C → I →; C → I →; C →
[23]	537 (M + F)	20 weeks	I - High-intensity strength exercise for neck/shoulders C - Guidelines and no treatment	Shoulders pain Neck pain	I ↓; C → I ↓; C →
[33]	20 (M + F)	16 weeks	I - Stretching exercises IC - Combined exercise and educational intervention EI - Educational intervention C - No treatment	Quality of life and health	I ↑; IC ↑; EI →; C →
[20]	66 (M + F)	10 weeks	I - Strength exercise for the upper body C - Ergonomic training	Work ability index Visual analog scale (	I ↑; C ↓ I ↓; C ↑
[21]	58 (F)	12 weeks	I - Hamstring stretching C – No treatment	ROM	I ↑; C: →
[25]	67 (NR)	12 weeks	I - Combination of aerobic and strength exercise C - No-treatment	Maximal oxygen uptake Isometric muscle strength Blood pressure Total cholesterol	I ↑; C → I →; C → I →; C → I →; C →
[34]	48 (M + F)	6 weeks	I - Yoga exercises C - No treatment	Profile of Mood States Bipolar Inventory of Positive Psychological Attitudes	I ↑; C → I ↑; C →
[35]	53 (M + F)	15 weeks	I - Light resistant training C - Exercise guidelines and no-treatment	Low back pain	I ↓; C →

*Abbreviations*: *I* intervention group; *C* control group; *1-RM* one-repetition maximum; *BMI* body mass index; *ROM* range of motion; *RPE* rates of perceived exertion; *IE* combined exercise and ergonomic modification group; *EI* educational intervention EG - ergonomic modification group; *IC* combined exercise and educational intervention; *EI* educational intervention; *ROM* range of motion; *M* male; *F* female; ↑ - increase; ↓ - decrease; → - no change; *NR* not reported

It appears that the majority of studies evaluated the effect of strength-stretching PA on various health domains, with resistance exercise interventions were employed most often. The duration and intensity of exercise interventions vary between 5 min and 20 min, from moderate- to high-intensity exercise, and the frequency of exercise was 3 to 5 times per week. Non-traditional programs favorably affected work performance, muscle-skeletal disorders, blood pressure, muscular and cardiorespiratory fitness, and mental health. Both high and moderate intensity exercise shown similar results. All studies reported rather high adherence to exercise, probably due to exercise-driven reduction in pain and improved health-related quality of life; non-traditional programs induced no side effects. Nevertheless, some interventions were more effective than others. Specifically, resistance exercise, either using free weights, body weight or the elastic band, was shown to be the most effective intervention, and at least 8 weeks are needed to achieve positive outcomes. In addition, the usage of mobile app with structured PA programs and contniuous monitoring resulted in higher adherence to exercise compared to traditional paper logs. Wearable technologies and mobile apps are recently identified as a hot topic in the fitness industry [30], and it would be interesting to see how technology affects future exercise programs at the workplace as well.

### Prescribing non-traditional exercise at workplace

We found a gap in the literature concerning the volume and intensity of non-traditional exercise programs performed at the worksite. There is no gold-standard for prescribing non-traditional PA interventions at workplace. Only a few studies examined this issue, concerning the volume, intensity and frequency of non-traditional programs at the workplace. Saeterbakken and colleagues [36] investigated the dose-response effect of resistance training for neck and shoulder pain relief at workplace. It appears that the daily bouts of specific high-intensity resistance training of the shoulder and neck could significantly decrease and prevent musculoskeletal disorders at the workplace. However, the authors did not find any differences between 10 min and 20 min exercise programs, suggesting no dose-response effect of this interevntion. Andersen et al. [37] found no difference in pain relief after either 60 min exercise once per week, 3 times per week of 20 min of exercise, and 7 times per week of 9 min of exercise among office workers. It appears that the total volume of PA is more important than the frequency of training sessions.

The majority of studies evaluated in this scientific forum follows the general recommendations of American College of Sports Medicine (ACSM) for exercise prescription [38]. A resistance-stretching exercise is often considered among the most effective tools for maintaining musculoskeletal fitness in workforce, with additional effects on health and well-being [2]. The general advice that puts forward 2–3 exercise sessions per week, recruiting large muscle groups for 8–12 repetitions per exercise set, could improve muscular fitness and health in general population, including workforce [1]. Practicing flexibility programs at least 2–3 times a week might complement resistance exercise while meeting individual needs and demands [39]. To conclude, approximately 15 min of non-traditional exercise at the workplace at least 3 times per week could improve health and well-being, decrease musculoskeletal pain, and advance work performance; this kind of intervention could be easily organized during short breaks at workplace. A minimum of 8-week intervention period is needed to see the benefitis, and low volume of moderate-to-high intensity exercise seem to be the most effective.

### Safety of non-traditional exercise at workplace

Taking part in exercise program could bring multiple health benefits yet several safety issues need to be considered. To ensure the safety of each PA program at the worplace, a preparticipation health survey remains a fundamental requisite. The survey usually identifies individuals with contraindications to exercise, individuals who should undergo a medical evaluation and exercise testing before starting the program, persons with clinically significant disease and other special needs. High-risk populations (e.g., obese and overweight people, active smokers, elderly, people who had a family history of heart disease) should consult a doctor before engaging in any PA program, including non-traditional exercise programs [38]. The diversity of workplace interventions reflects a variety of potential hazards. Pre-exercise protocols, such as ensuring an adequate place for exercise, proper equipment, and exercise specialist to guide and supervise the program, could decrease the risk of workplace exercise interventions [40]. Non-traditional exercise programs are considered safe in comparison to collective sport activities [41], with no major adverse effects reported throughout the previous studies. Nevertheless, the employees who intend to participate in workplace programs are strongly advise to gradually increase the volume and intensity of exercise. Warm-up activities might be recommended to avoid any adverse outcomes [42, 43], with moderate intensity PA considered generaly safe for workplace-based exercise interventions.

## Conclusion

Non-traditional PA programs at the workplace appears to be associated with improved health outcomes, and resistance exercise has been found to be superior to other interventions. A brief intervention of 15 min per day at least 3 times per week for over 8 weeks can reduce musculoskeletal pain and improve work performance. Non-traditional exercise interventions produces no major side effects, with minimum risk of exercise-induced injuries. Taking part in non-traditional exercise can improve health-related physical fitness and prevent sedentary behavior at the workplace, and those innovative programs might be of utmost importance for healthy lifestyle promotion in this sensible population.

## Data Availability

Not applicable.

## Abbreviations

ACSM: American College of Sports Medicine
PA: Physical activity
SPORT4H: Sport for Health Network
WHO: World Health Organization
